# Variation in insulative feather structure in songbirds replacing each other along a tropical elevation gradient

**DOI:** 10.1002/ece3.8698

**Published:** 2022-03-10

**Authors:** Sahas Barve, Carlos Daniel Cadena

**Affiliations:** ^1^ Smithsonian National Museum of Natural History Washington District of Columbia USA; ^2^ 27991 Departamento de Ciencias Biológicas Universidad de los Andes Bogotá Colombia

**Keywords:** eco‐physiology, elevational distribution, *Henicorhina*, mountains, museum collections, natural history

## Abstract

High‐elevation organisms are expected to evolve physiological adaptations to cope with harsh environmental conditions. Yet, evidence for such adaptive differences, especially compared to closely related lowland taxa occurring along the same elevational gradient, is rare. Revisiting an anecdotal natural history observation by O. Bangs from 1899 and based on new measurements of museum specimens, we confirmed that the high‐elevation hermit wood wren (*Henicorhina anachoreta*) from the Sierra Nevada de Santa Marta, Colombia, has longer, more insulative feathers on the chest and back, than its lower‐elevation counterpart the grey‐breasted wood wren (*H*. *leucophrys*). However, we did not find evidence for the same specializations in subspecies of *H*. *leucophrys* that live at high elevations on other elevational gradients in the Colombian Andes, although similar adaptive solutions have arisen in separate mountain systems like the Himalayas. Adaptations in plumage may be associated with the recurrence of elevational species replacements throughout the tropics.

## Graphical abstract

We show that high‐elevation *H*. *anachoreta* has more insulative feathers than low‐elevation *H*. *leucophrys*. But *H*. *leucophrys* has not evolved similar modification in regions where it has colonized high elevations.

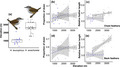

## 1 INTRODUCTION

How do tropical organisms living at high elevations cope with the demands imposed by such harsh environments? Answering this question is crucial to understand the elevational range limits of species and to forecast their responses to climatic change (Jankowski et al., 2013; Linck et al., 2021). An example of the challenges posed by living at high elevations is the reduced partial pressure of oxygen, which has prompted numerous physiological adaptations in vertebrates (Storz, 2021). Although hematological adaptations to high elevation in birds have been well characterized (e.g., Barve et al., 2016), how birds cope with low temperatures at high elevations has been much less studied and the mechanisms conferring tolerance to cold conditions in tropical species are not well understood (Londoño et al., 2017).

A likely axis along which bird species from tropical mountains may adapt to cold temperatures in the highlands is via increased insulation provided by their plumage (Gamero et al., 2015). For example, in the original taxonomic description of the hermit wood wren (Troglodytidae, *Henicorhina anachoreta*) from the Sierra Nevada de Santa Marta, northern Colombia, Outram Bangs outlined an intriguing adaptive hypothesis. When comparing the high‐elevation taxon *anachoreta* to parapatric populations from lower elevations, now referable to his namesake grey‐breasted wood wren (*H*. *leucophrys bangsi*, see Caro et al., 2013; Cadena et al., 2016), Bangs (1899) subjectively noted that plumage was “everywhere, much longer and denser, but especially noticeably so on the head and neck, the bird evidently being fitted to withstand cold weather.” The possibility that birds from higher elevations in Santa Marta may have plumage adapted to cold temperatures is tantalizing given their smaller body size compared to birds from lower elevations (Cadena et al., 2016), which is contrary to the prediction that endotherms should increase relative body surface area in cold environments to reduce heat loss through thermal conductance to the environment (i.e., Bergmann's rule; Freeman, 2017).

The role of plumage providing insulation has long been recognized in studies of climatic adaptation in birds (Scholander, 1955) and several examples point to variation in feather structure associated with living in cold environments (de Zwaan et al., 2017; Koskenpato et al., 2016; Pap et al., 2020). In particular, a recent study documented a widespread increase in the downy section of contour feathers and in contour feather length with elevation in passerine birds from the Himalayas, revealing patterns of convergent evolution resulting in more insulative feathers and plumage at higher elevations across several passerine families (Barve et al., 2021). The proportion of down increases insulative capacity in each feather, whereas longer feathers give rise to a deeper, more insulative overall plumage (Pap et al., 2017). Barve et al. (2021) showed that Himalayan taxa may show one or both traits along elevational gradients. However, not all species examined showed variation in feather structure across the elevational gradient (Barve et al., 2021), suggesting that plumage modifications may or may not arise as a result of the effect of factors such as a species’ metabolic flexibility (Londoño et al., 2015), gene flow across the elevational gradient swamping adaptive variation (Bridle et al., 2009), or plasticity or lack thereof in the trait itself (Price et al., 2003).

Elevational replacement of closely related bird species is a pervasive pattern in tropical mountains across the world (reviewed by Cadena & Céspedes, 2020). Several ecological hypotheses have been proposed to account for such patterns of replacement, with a common conjecture being that the physiological stress of cold temperatures, at least in part, sets the upper elevational limit of the low‐elevation species, where it is replaced by its high‐elevation counterpart (Barve & Dhondt, 2017; Freeman et al., 2019; Terborgh, 1977). However, case studies where adaptation to cold temperature is demonstrated in the highland species in an elevationally replacing species pair are rare (Jankowski et al., 2013).

**FIGURE 1:**
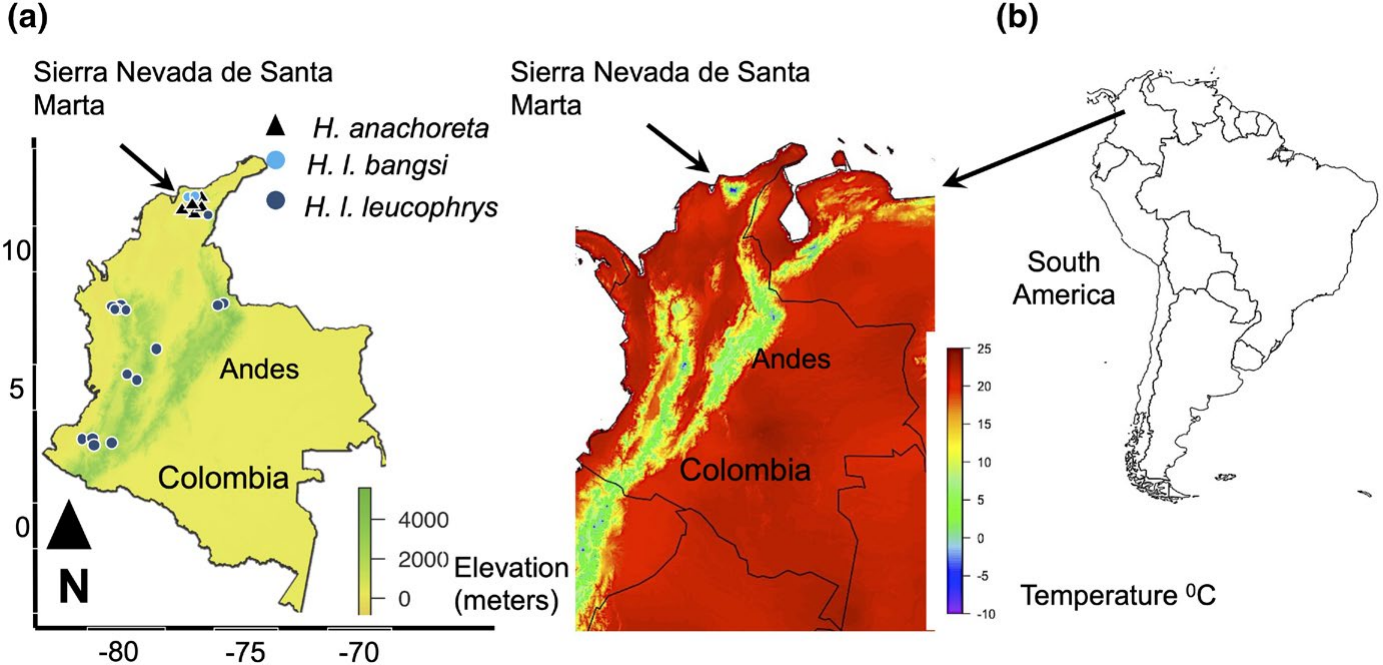
(a) Map showing the location of the specimens used in the study. Black triangles and light blue circles show the collection locations in the Sierra Nevada de Santa Marta mountains disjunct from the Andes. Specimens of other subspecies of *H*. *leucophrys* were collected throughout the Colombian Andes (dark blue circles, locations derived from Paynter et al. (1997)). (b) Data on mean minimum temperature in Colombia between 1971 and 2000 indicate the Sierra Nevada de Santa Marta and Andes have similar patterns of temperature variation with elevation. Temperature data were downloaded from WorldClim 2.1 (Fick & Hijmans, 2017). The map was generated using the R packages *ggplot2* (Wickham, 2016), *sf* (Pebesma, 2018), *maps* (Brownrigg, 2013), and *elevatr* (Hollister et al., 2017)

The Sierra Nevada de Santa Marta is widely regarded as the tallest coastal mountain on Earth, rising from sea level in the hot Caribbean lowlands to snow‐covered peaks corresponding to the highest elevations in Colombia (Figure 1a). Wood wren species in the genus *Henicorhina* replace each other with elevation in Santa Marta (Caro et al., 2013; Todd & Carriker, 1922), with *H*. *leucophrys* occurring in warm forest environments typically below 2000 m and *H*. *anachoreta* in colder cloud forests and elfin forests reaching more than 3000 m (see Cleef et al. (1984) for details on climatic and ecological variation with elevation in the region). The steep temperature gradients existing along mountain slopes in the Sierra Nevada de Santa Marta (Figure 1b) may have a strong impact on the thermal biology of organisms; for example, the mean activity temperatures of frog species studied at sites at 1500 and 3500 m in the region differ by more than 8°C (Rueda Solano et al., 2016). We investigated whether the plumage of elevationally replacing wood wrens varies adaptively in the context of thermoregulation in the Sierra Nevada de Santa Marta. We first tested whether wood wrens show clinal variation in feather traits, with individuals from lower elevations within each species showing less insulative feathers compared to conspecific individuals from higher elevations. Next, we tested whether the pattern described subjectively by Bangs (1899) is verified by quantitative analyses focused on variables reflecting the insulative properties of feathers, that is, whether the higher‐elevation species *H*. *anachoreta* has more insulative plumage than the lower‐elevation species *H*. *leucophrys*.

On elevational gradients in the Colombian Andes exhibiting similar temperature variation to that existing in the Sierra Nevada de Santa Marta (Figure 1b), various forms of *H*. *leucophrys* reach high elevations in the absence of *H*. *anachoreta*. We examined whether individuals of *H*. *leucophrys* from the Andes, which live higher than *H*. *l*. *bangsi,* show increased thermo‐insulative feathers. We thus tested whether Andean specimens from above 1500 m of subspecies *H*. *l*. *leucophrys*, *H*. *l*. *manastarae*, and *H*. *l*. *brunneiceps,* showed significantly increased proportion of down or relative feather length compared to the lower‐elevation *H*. *l*. *bangsi*.

## 2 METHODS

### 2.1 Feather structure measurements

We studied all specimens of *Henicorhina leucophrys bangsi* (*N* = 15) and *Henicorhina anachoreta* (*N* = 37) in the collection of the Smithsonian National Museum of Natural History, Washington D.C., USA (NMNH), collected within the Sierra Nevada de Santa Marta (Figure 1a) with either a definite elevation of collection or elevation recorded as within a range of ~300 m (1000 feet) for which we assumed the midpoint of the range as the elevation of collection. We also studied specimens with known elevation of *H*. *leucophrys* subspecies found above 1500 m in other Colombian mountains (*H*. *leucophrys brunneiceps*, *N* = 3, *H*. *leucophrys leucophrys*, *N* = 44, and *H*. *leucophrys manastarae*, *N* = 2). Following the non‐destructive protocol used in Barve et al. (2021) and because of Bangs’ observations (Bangs, 1899), we measured one feather on the chest and one feather at the base of the neck (upper back, henceforth back feathers) of each specimen. Briefly, we photographed a feather using the Leica Application Suite, LAS V2.6 image analysis software, with image calibration for measuring length millimeters (Leica Microsystems, Wetzlar Germany). Using these images, we measured (1) length of plumulaceous or downy section of the feather, the downy section was designated as the region with non‐interlocking fluffy‐textured barbs; (2) length of pennaceous section, the region of the feather with interlocking barbs; and (3) total length of feather, the sum of the downy and pennaceous sections.

### 2.2 Statistical analyses

For 34 specimens (10 each of *H*. *anachoreta*, *H*. *l*. *bangsi*, and *H*. *l*, *leucophrys*, and 2 each of *H*. *l*. *brunneiceps and H*. *l*. *manastarae*; i.e., 33% of all specimens used in the study), we ensured repeatability of all feather structure measurements described above by measuring the same variables on two adjacent feathers and using the R package *Caper* to estimate the interclass correlation coefficient (Orme et al., 2013). Both feather measurements had high repeatability (interclass correlation coefficient) within an individual (proportion of downy section: chest = 0.87, back = 0.83; and total feather length: chest = 0.90, back = 0.89).

Because *H*. *anachoreta* is smaller than *H*. *leucophrys* (Cadena et al., 2016), we controlled for body size in feather length measurements by dividing the total feather length by the median body mass of the species measured in an earlier study (Caro et al., 2013). While testing for differences between high‐ and low‐elevation specimens of *H*. *leucophrys*, we corrected for differences in body size by dividing the total feather length by the tarsus length of the specimen. Therefore, all analyses were done using measures of relative feather length. To reduce redundancy in our analyses, we first confirmed that there was little correlation between proportion of downy section and feather length for the chest (*r* = .14, *p* = .15) or back feathers (*r* = .03, *p* = .33). Next, we used these two variables to test for variation in feather structure with elevation in the two elevationally replacing species from the Sierra Nevada de Santa Marta using linear multiple regressions. In each multiple regression, the feather structure variable (either proportion down or feather length) was the dependent variable and an interaction of the species ID and elevation was the independent variable. Through this analysis, we wanted to explore whether one or both species showed within‐species variation in feather structure with elevation (as a likely response to temperature) and whether the slopes of the relationships between elevation and feather structure varied between species.

We also used multiple regressions to test for differences in proportion of downy section and feather length in the lowland *H*.*l*. *bangsi* and highland specimens of *H*. *leucophrys* collected from other regions of its range. In all cases, we did the analyses separately for chest and back feathers. All analyses were done in the R Statistical Programming Language 4.0.5 (R Core Team, 2021).

## 3 RESULTS

**FIGURE 2:**
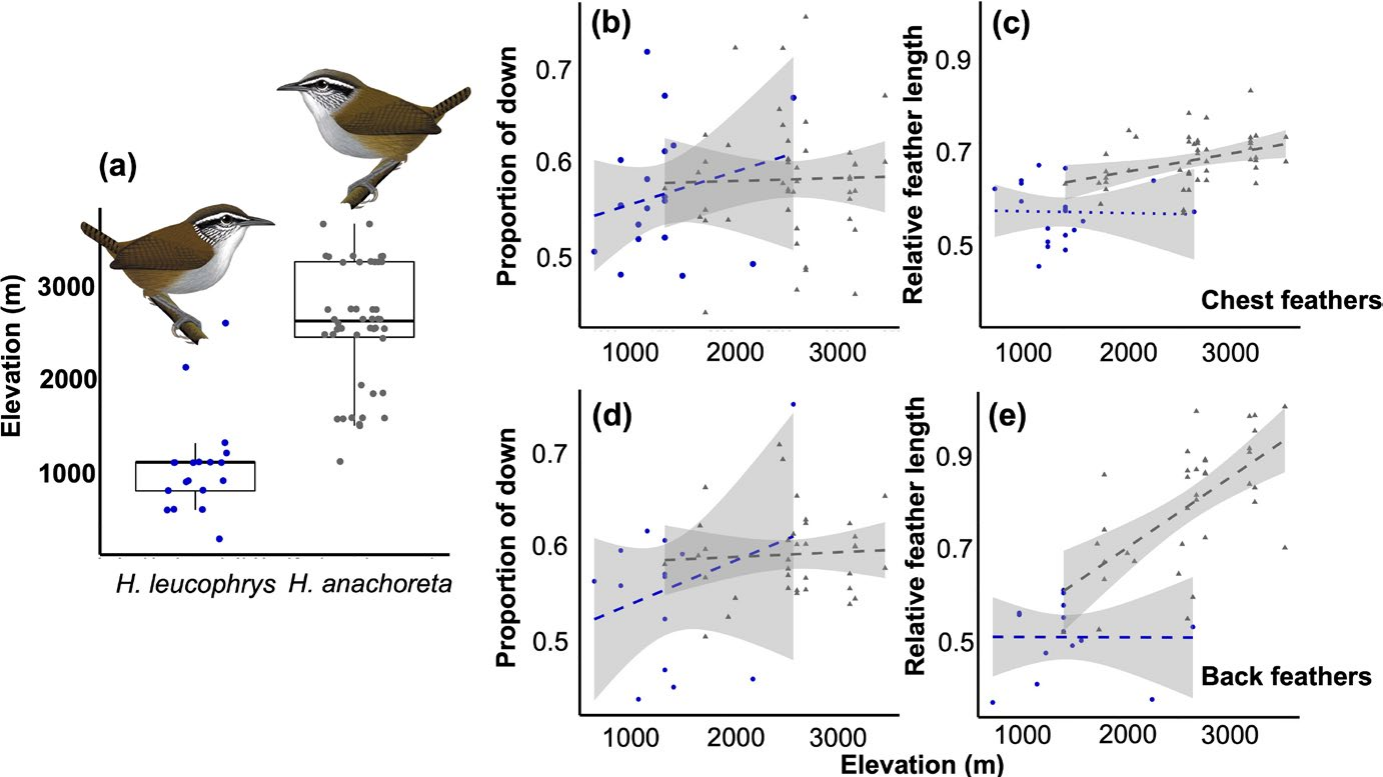
Feather structure variation in *H*. *leucophrys bangsi* (blue dots) and *H*. *anachoreta* (grey triangles) along the Sierra Nevada de Santa Marta is consistent with adaptation to varying temperatures. (a) *H*. *leucophrys*, a largely low‐ and mid‐elevation species, is replaced by *H*. *anachoreta* at high elevation as shown by data on study specimens. (b–e) Lines represent predicted linear regression lines, and shaded areas are standard errors for *H*. *leucophrys* (blue dots and line) and *H*. *anachoreta* (grey triangles and line). On the chest, (b) proportion of downy section did not increase with elevation in either species. (c) High‐elevation wood wrens had longer feathers in general but the slopes of increase were not significantly different between species. On the back feathers, (d) proportion of downy section did not increase with elevation but (e) feather length increased with elevation, overall feather length was shorter in the low‐elevation *H*. *leucophrys*, and feather length increased more steeply with elevation in the high‐elevation *H*. *anachoreta*. Artwork reproduced with permission from the author (Ayerbe‐Quiñones, 2018)

**FIGURE 3:**
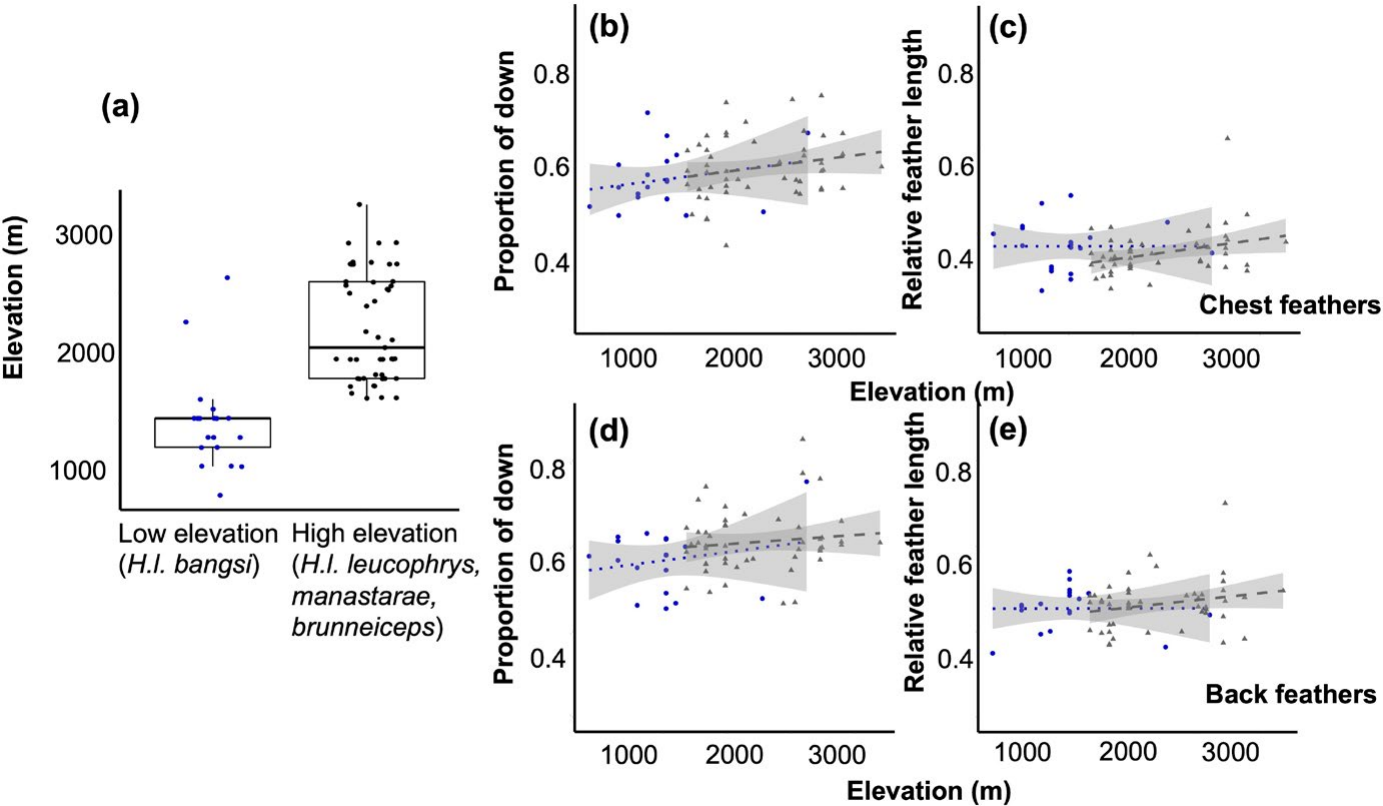
(a) There is no significant difference between the feather structure in *H*. *leucophrys bangsi* (blue dots) and highland taxa of *H*. *leucophrys* (black dots). (b–e) Lines represent predicted linear regression lines, shaded areas are standard errors for low‐elevation *H*. *leucophrys* (blue dots and line) and high‐elevation *H*. *leucophrys* (grey triangles and line). Raw data points are shown. (a) Elevational distribution of specimens used in the study. On the chest feathers, (b) and (c), proportion of downy section and relative feather length was not significantly different. On the back feathers, (d) and (e), proportion of downy section and relative feather length was also not significantly different

Patterns of variation in feathers within and between species along the elevational gradient in the Sierra Nevada de Santa Marta were consistent with adaptive plumage scenarios given the occurrence of specimens of *H*. *leucophrys* at elevations lower than those of *H*. *anachoreta* (Table 1; Figure 2a). On the chest feathers, the proportion of downy section did not vary with elevation in either species, but the highland *H*. *anachoreta* had overall longer feathers relative to body size (*p* = .03, Table 1, Figure 2c). On the back, as on the chest, the proportion of the downy section in feathers did not change with elevation. However, *H*. *anachoreta* had significantly longer back feathers (*p* < .001, Table 1, Figure 2e) than *H*. *leucophrys*, the low‐elevation species. Relative feather length increased with elevation significantly more steeply in *H*. *anachoreta* than in *H*. *leucophrys* for back feathers (significant interaction term between elevation and species, *p* = .02, Table 1, Figure 2e). On both the chest and the back, the low‐elevation *H*. *l*. *bangsi* tended to have more variation in feather structure values than *H*. *anachoreta* (Figure 2), or the high‐elevation specimens of *H*. *leucophrys* from the Andes (Figure 3). In contrast to our results from the Sierra Nevada de Santa Marta, when extending our analyses to other elevational gradients, we found no statistical difference in feather structure on the chest feathers (Figure 3b,c, Table 2) or back feathers (Figure 3d,e, Table 2) between the lowland *H*. *l*. *bangsi* and three highland Andean taxa within *H*. *leucophrys*.

**TABLE 1:** Result of linear multiple‐regression models testing the role of an interaction between elevation and species in explaining the variation in feather structure in wood wrens from the Sierra Nevada de Santa Marta. Significant differences (*p* < .05) are highlighted. *Henicorhina anachoreta* was the reference level and compared to *H*. *leucophrys bangsi* in all models

Chest feathers	Proportion of downy section ~ Elevation × Species (*R* ^2^ = 0.01, *p* = .60)			
	Estimate	*SE*	*t*	*p*
Elevation	3.5 × 10^−6^	2.05 × 10^−5^	0.17	.86
Species (*H*. *leucophrys*)	−6.7	8.18 × 10^−2^	0.8	.42
Elevation × Species (*H*. *leucophrys*)	3.57 × 10^−5^	4.48 × 10^−5^	0.79	.42

Chest feathers	Relative feather length ~ Elevation × Species (*R* ^2^ = 0.47, *p* < .001)			
	Estimate	*SE*	*t*	*p*
Elevation	4.8 × 10^−5^	1.9 × 10^−5^	2.57	.**01**
Species (*H*. *leucophrys*)	0.01	0.07	0.13	.89
Elevation × Species (*H*. *leucophrys*)	5.2 × 10^−5^	0.4 × 10^−5^	−1.2	.19

Back feathers	Proportion of downy section ~ Elevation × Species (*R* ^2^ = 0.04, *p* < .12)			
	Estimate	*SE*	*t*	*p*
Elevation	3.1 × 10^−6^	1.8 × 10^−5^	0.17	.85
Species (*H*. *leucophrys*)	0.08	0.07	−1.2	.23
Elevation × Species (*H*. *leucophrys*)	3.6 × 10^−5^	3.73 × 10^−5^	0.96	.34

Back feathers	Relative feather length ~ Elevation × Species (*R* ^2^ = 0.63, *p* < .001)			
	Estimate	*SE*	*t*	*p*
Elevation	1.2 × 10^−4^	2.2 × 10^−5^	4.9	**<.001**
Species (*H*. *leucophrys*)	−0.12	0.09	1.3	.18
Elevation × Species (*H*. *leucophrys*)	−1.2 × 10^−4^	5.0 × 10^−5^	2.44	.**02**

**TABLE 2:** Results of linear multiple‐regression testing the role of taxon (low/high) within *H*. *leucophrys* in explaining the variation in feather structure. The low‐elevation *H*. *l*. *bangsi* is used as the reference level in all models and compared to high‐elevation specimens of *H*. *leucophrys*

Chest feathers	Proportion of downy section ~ Elevation × Species (*R* ^2^ = 0.03, *p* = .15)			
	Estimate	*SE*	*t*	*p*
Elevation	3.8 × 10^−6^	2.4 × 10^−5^	0.16	.36
Species *(H*. *leucophrys*)	1.2 × 10^−2^	8.5 × 10^−2^	0.01	.98
Elevation × Species (*H*. *leucophrys*)	1.1 × 10^−6^	4.9 × 10^−5^	0.02	.98

Chest feathers	Relative feather length ~ Elevation × Species (*R* ^2^ = 0.02, *p* = .17)			
	Estimate	*SE*	*t*	*p*
Elevation	3.9 × 10^−5^	2.1 × 10^−5^	1.92	.90
Species (*H*. *leucophrys*)	0.01	0.07	0.15	.14
Elevation × Species (*H*. *leucophrys*)	3.8 × 10^−5^	0.4 × 10^−5^	−0.93	.35

Back feathers	Proportion of downy section ~ Elevation × Species (*R* ^2^ = 0.04, *p* = .13)			
	Estimate	*SE*	*t*	*p*
Elevation	2.2 × 10^−5^	2.53 × 10^−5^	0.84	.4
Species (*H*. *leucophrys*)	0.05	0.09	−0.59	.55
Elevation × Species (*H*. *leucophrys*)	1.7 × 10^−5^	5.19 × 10^−5^	0.33	.73

Back feathers	Relative feather length ~ Elevation × Species (*R* ^2^ = 0.003, *p* = .36)			
	Estimate	*SE*	*t*	*p*
Elevation	3.2 × 10^−5^	1.91 × 10^−5^	1.68	.90
Species (*H*. *leucophrys*)	−0.06	0.06	0.88	.37
Elevation × Species (*H*. *leucophrys*)	3.2 × 10^−5^	3.9 × 10^−5^	0.8	.42

## 4 DISCUSSION

Our quantitative analyses of feather structure based on museum specimens allowed us to confirm Bangs’ (1899) anecdotal observation that high‐elevation *Henicorhina* wood wrens from the Sierra Nevada de Santa Marta may have longer, more insulative plumage than their lower‐elevation counterparts to cope with cooler temperatures. Himalayan birds increase the insulation provided by their plumage via increasing the proportion of down in their feathers or by increasing the length of their feathers, which thus overlap more extensively with neighboring feathers to produce a denser, more insulative plumage (Barve et al., 2021). We found that *Henicorhina* wood wrens in Santa Marta seemingly use this second strategy, in agreement with Bangs’ (1899) hypothesis.

We did not find a significant increase in the proportion of down in either the chest or back contour feathers, but did see relatively longer feathers at higher elevations in both regions of the body in the high‐elevation species *H*. *anachoreta* from Santa Marta. The high‐elevation *H*. *anachoreta* also showed a steeper change in back feather length with elevation than its lowland congener, suggesting that thermo‐insulation through plumage is likely more important in the cold, high elevations of the Sierra Nevada de Santa Marta (Figure 2c,e). Based on the width of the error bands around linear regressions, low‐elevation *H*. *leucophrys* specimens also showed greater variation in feather variables than the highland *H*. *anachoreta* or the highland specimens of *H*. *leucophrys* from the Andes. This suggests that there is potentially a greater cost for variation in feather structure at higher elevations where insulative structures are more important. Such a result is in agreement with the idea that cold temperatures and other harsh conditions may act as environmental filters restricting the diversity of phenotypes existing within and among species at high elevations in tropical mountains (Graham et al., 2012).

Elevational species replacement is a ubiquitous global pattern in many groups of organisms, particularly in tropical mountains (Cadena & Céspedes, 2020). Although lower‐elevation species are often competitively superior to high‐elevation taxa (Jankowski et al., 2010; but see Freeman, 2020), the upper elevational range limits of species from lower elevations may be set by physiological constraints, restricting their elevational ranges, and allowing their coexistence in the same mountain slopes with species better adapted to the harsh conditions of high elevations. Nevertheless, evidence for differences in phenotypic characters that may directly confer a physiological advantage to high‐elevation species is rare (Gaffney, 2017). Multiple factors determine the elevational distribution of species (Sexton et al., 2009), but temperature is an important factor, especially for small endotherms like birds (Elsen et al., 2016; Tingley et al., 2009). Our results show that in the elevationally replacing *Henicorhina* wood wrens of the Sierra Nevada de Santa Marta, the highland *H*. *anachoreta* indeed has more insulative plumage than the low‐elevation *H*. *leucophrys*, thus pointing to a morphological axis along which *H*. *anachoreta* may be more specialized to live at high, cool elevations than *H*. *leucophrys*. In turn, *H*. *leucophrys* appears to be behaviorally dominant over *H*. *anachoreta* as indicated by song playback experiments revealing asymmetric responses between species to simulated interspecific territorial intrusions (Burbidge et al., 2015). Whether the patterns we documented indeed result in increased thermoregulatory costs restricting *H*. *leucophrys* to low elevations and enabling the persistence of *H*. *anachoreta* due to physiological adaptations at higher elevations awaits experimental confirmation. However, we note that other high‐elevation birds from the Neotropics do loose less heat to the environment compared to their counterparts from lower elevations, in part because of plumage (Londoño et al., 2017).

We found that variation in feather structure with elevation in Santa Marta is clinal, both between species and within species (especially in *H*. *anachoreta*), with a continuous trend of increasing relative feather length and considerable overlap in measurements between species. Such patterns contrast with work showing that *H*. *leucophrys* and *H*. *anachoreta* are distinctly different in several morphological measurements describing bill size and shape and body size, and show no clear pattern of within‐species variation in such morphological traits along their elevational distribution in Santa Marta (Caro et al., 2013). Assuming that such bill and body dimensions are strongly heritable, the fact that feather structure varies more gradually indicates that this trait may be more plastic, dependent more on the elevation individuals live at, rather than on species‐specific genetic factors.

Populations in the *H*. *leucophrys* complex occurring at high elevations in the Sierra Nevada de Santa Marta (Caro et al., 2013) and the Andes of Ecuador (Dingle et al., 2008, 2010) have seemingly converged in attributes of their song and morphology, a situation which may also occur in the Cordillera de Mérida in Venezuela (J. L. Pérez‐Emán, unpubl. data). In contrast, our results showing no differences in feather structure between *H*. *l*. *bangsi* and allopatric taxa in *H*. *leucophrys* which extend into the highlands, reveals that the seemingly adaptive pattern observed in *H*. *anachoreta* in Santa Marta has not arisen in other elevational gradients in the Andes despite exhibiting similar thermal gradients. Moreover, we believe such results may also offer a hint as to the potential mechanisms underlying the variation we observed in Santa Marta. Assuming that a similar genetic architecture underlies phenotypic variation in feather structure in different populations of wood wrens, we hypothesize that the lack of elongated feathers at cool, high elevations in *H*. *leucophrys* in the Andes outside of Santa Marta implies that modifications observed in *H*. *anachoreta* in Santa Marta are not entirely induced by the environment as one may have speculated based on our discussion of clinal variation in the paragraph above.

If modifications in feathers associated with elevation are at least partly under genetic control in *H*. *anachoreta*, then why have they not arisen in other populations of *H*. *leucophrys* which experience similarly cold temperatures at high elevations? Three relatively simple explanations are that (1) mutations encoding adaptive phenotypes have not arisen in other populations, (2) despite overall similarities in climatic gradients between the Santa Marta and the Andes, regional differences in factors other than, or in addition to temperature, have led to an absence of convergent evolution in feathers; or (3) populations from different regions may have responded to the challenges of high elevations in different ways (e.g., more insulative plumage vs. greater thermogenic capacity). Alternatively, we speculate that in mountains where a single species exists across a large elevational gradient, high gene flow along mountain slopes (as shown in Linck et al., 2020 and Pujolar et al., 2022, but see Polato et al., 2018) may restrict the emergence of adaptive variation in the highlands owing to swamping of locally beneficial alleles by maladaptive variants arriving via dispersal from lower elevations (Bachmann et al., 2020; Bridle et al., 2009; Bridle & Vines, 2007; Polechová & Barton, 2015). In contrast, because *H*. *anachoreta* is reproductively isolated from its lower‐elevation counterpart *H*. *l*. *bangsi*, absence of gene flow from the lowlands may have facilitated adaptation. In any case, additional work assessing the heritability and plasticity of feather attributes (e.g., common garden experiments) is necessary to understand how feather traits presumably influencing organismal performance evolve.

In conclusion, elevationally replacing wood wrens in the Sierra Nevada de Santa Marta (but not in the Andes) showed variation in feather structure with elevation similar to that shown by phylogenetically distant Himalayan birds. This suggests that feather modifications that increase their thermo‐insulative potential may be a widespread adaptation in the world's montane birds. Expanding our study to a larger set of South American birds would allow to further confirm this generality. Our work underscores the immense value of museum collections in the comparative research of morphological trait evolution. Natural history collections provide critical access to biological materials where newly developed techniques can be used to comprehensively test classical eco‐evolutionary hypotheses.

## CONFLICT OF INTEREST

The authors have no conflict of interest.

## AUTHOR CONTRIBUTIONS

**Sahas Barve:** Conceptualization (equal); Data curation (lead); Formal analysis (lead); Investigation (equal); Methodology (lead); Project administration (equal); Visualization (equal); Writing – original draft (equal); Writing – review & editing (equal). **Carlos Daniel Cadena:** Conceptualization (lead); Investigation (equal); Writing – original draft (equal); Writing – review & editing (equal).

## Supporting information

Supplementary Material

## Data Availability

Data are made available as supplementary material.
