# Supplementary material for: Variation in insulative feather structure in songbirds replacing each other along a tropical elevation gradient
Source: Ecol Evol. 2022 Mar 10;12(3):e8698. doi: 10.1002/ece3.8698 (PMC8928881; doi:10.1002/ece3.8698)
Supplement: Supplementary file 1 — Supplementary Material [file ECE3-12-e8698-s001.pdf]

USNM#	SPECIES	Side	TLBODYMASS	PDL	METERS
384093	H. anachoreta	Dorsal	0.54	0.59	1515.15
384093	H. anachoreta	Ventral	0.50	0.53	1515.15
387749	H. anachoreta	Ventral	0.57	0.51	1863.64
387749	H. anachoreta	Dorsal	0.64	0.52	1863.64
387750	H. anachoreta	Dorsal	0.77	0.61	1863.64
387750	H. anachoreta	Ventral	0.53	0.59	1863.64
387751	H. anachoreta	Ventral	0.51	0.72	2727.27
387751	H. anachoreta	Dorsal	0.79	0.63	2727.27
387752	H. anachoreta	Dorsal	0.80	0.58	3106.06
387752	H. anachoreta	Ventral	0.61	0.54	3106.06
387753	H. anachoreta	Dorsal	0.87	0.59	3409.09
387753	H. anachoreta	Ventral	0.61	0.56	3409.09
387754	H. anachoreta	Dorsal	0.74	0.56	2651.52
387754	H. anachoreta	Ventral	0.61	0.42	2651.52
387755	H. anachoreta	Dorsal	0.84	0.62	3151.52
387755	H. anachoreta	Ventral	0.56	0.53	3151.52
387756	H. anachoreta	Dorsal	0.79	0.57	2727.27
387756	H. anachoreta	Ventral	0.58	0.57	2727.27
387757	H. anachoreta	Ventral	0.66	0.45	2727.27
387757	H. anachoreta	Dorsal	0.77	0.58	2727.27
387758	H. anachoreta	Ventral	0.53	0.44	2727.27
387758	H. anachoreta	Dorsal	0.77	0.64	2727.27
387760	H. anachoreta	Dorsal	0.73	0.57	2651.52
387760	H. anachoreta	Ventral	0.58	0.47	2651.52
387761	H. anachoreta	Ventral	0.62	0.49	3151.52
387761	H. anachoreta	Dorsal	0.73	0.56	3151.52
387762	H. anachoreta	Dorsal	0.68	0.61	2727.27
387762	H. anachoreta	Ventral	0.56	0.60	2727.27
387763	H. anachoreta	Dorsal	0.79	0.51	3106.06
387763	H. anachoreta	Ventral	0.59	0.50	3106.06
387763	H. anachoreta	Dorsal	0.86	0.55	3106.06
387763	H. anachoreta	Ventral	0.56	0.57	3106.06
387764	H. anachoreta	Dorsal	0.66	0.66	3409.09
387764	H. anachoreta	Ventral	0.56	0.63	3409.09
387765	H. anachoreta	Ventral	0.58	0.54	2651.52
387765	H. anachoreta	Dorsal	0.78	0.61	2651.52
387766	H. anachoreta	Dorsal	0.75	0.61	3151.52
387766	H. anachoreta	Ventral	0.57	0.42	3151.52
387766	H. anachoreta	Dorsal	0.70	0.61	3151.52
387766	H. anachoreta	Ventral	0.50	0.56	3151.52
387767	H. anachoreta	Dorsal	0.81	0.57	3106.06
387767	H. anachoreta	Ventral	0.72	0.52	3106.06
387767	H. anachoreta	Dorsal	0.78	0.60	3106.06
387767	H. anachoreta	Ventral	0.60	0.54	3106.06

387769	H. anachoreta	Dorsal	0.86	0.61	3151.52
387769	H. anachoreta	Ventral	0.54	0.56	3151.52
387770	H. anachoreta	Ventral	0.60	0.53	3106.06
387770	H. anachoreta	Dorsal	0.76	0.63	3106.06
387771	H. anachoreta	Dorsal	0.87	0.66	2651.52
387771	H. anachoreta	Dorsal	0.82	0.59	2651.52
387771	H. anachoreta	Ventral	0.51	0.49	2651.52
387771	H. anachoreta	Ventral	0.60	0.55	2651.52
387772	H. anachoreta	Dorsal	0.67	0.60	1803.03
387772	H. anachoreta	Ventral	0.51	0.55	1803.03
387773	H. anachoreta	Dorsal	0.54	0.63	1818.18
387773	H. anachoreta	Ventral	0.46	0.50	1818.18
387774	H. anachoreta	Dorsal	0.65	0.54	2060.61
387774	H. anachoreta	Dorsal	0.74	0.55	2060.61
387774	H. anachoreta	Ventral	0.63	0.58	2060.61
387774	H. anachoreta	Ventral	0.54	0.50	2060.61
387775	H. anachoreta	Ventral	0.52	0.40	1863.64
387775	H. anachoreta	Dorsal	0.69	0.58	1863.64
387776	H. anachoreta	Dorsal	0.62	0.67	1863.64
387776	H. anachoreta	Ventral	0.49	0.50	1863.64
387776	H. anachoreta	Ventral	0.51	0.56	1863.64
387777	H. anachoreta	Dorsal	0.56	0.59	2575.76
387777	H. anachoreta	Dorsal	0.64	0.59	2575.76
387777	H. anachoreta	Ventral	0.53	0.56	2575.76
387777	H. anachoreta	Ventral	0.53	0.53	2575.76
387778	H. anachoreta	Dorsal	0.72	0.62	2575.76
387778	H. anachoreta	Ventral	0.55	0.56	2575.76
387779	H. anachoreta	Ventral	0.67	0.51	2575.76
387779	H. anachoreta	Ventral	0.60	0.56	2575.76
387779	H. anachoreta	Dorsal	0.78	0.58	2575.76
387780	H. anachoreta	Ventral	0.61	0.53	2575.76
387780	H. anachoreta	Dorsal	0.81	0.57	2575.76
387789	H. anachoreta	Ventral	0.61	0.68	2121.21
387789	H. anachoreta	Dorsal	0.64	0.56	2121.21
387790	H. anachoreta	Dorsal	0.77	0.60	2575.76
387790	H. anachoreta	Ventral	0.49	0.58	2575.76
387792	H. anachoreta	Dorsal	0.72	0.55	2530.30
387792	H. anachoreta	Ventral	0.43	0.54	2530.30
387793	H. anachoreta	Ventral	0.54	0.62	2500.00
387793	H. anachoreta	Dorsal	0.62	0.71	2500.00
387793	H. anachoreta	Dorsal	0.52	0.66	2500.00
387794	H. anachoreta	Dorsal	0.59	0.62	2621.21
387794	H. anachoreta	Ventral	0.60	0.53	2621.21
387795	H. anachoreta	Ventral	0.49	0.68	2530.30
387795	H. anachoreta	Ventral	0.44	0.60	2530.30

387795	H. anachoreta	Dorsal	0.67	0.70	2530.30
170348	H.l.bangsi	Ventral	0.51	0.45	2272.73
170348	H.l.bangsi	Dorsal	0.44	0.48	2272.73
170349	H.l.bangsi	Dorsal	0.43	0.58	909.09
170349	H.l.bangsi	Ventral	0.49	0.46	909.09
253449	H.l.bangsi	Ventral	0.40	0.51	1363.64
253449	H.l.bangsi	Dorsal	0.51	0.63	1363.64
374499	H.l.bangsi	Dorsal	0.56	0.57	1136.36
374499	H.l.bangsi	Ventral	0.46	0.56	1136.36
374500	H.l.bangsi	Ventral	0.55	0.49	1287.88
374500	H.l.bangsi	Ventral	0.31	0.48	1287.88
374500	H.l.bangsi	Dorsal	0.54	0.55	1287.88
374500	H.l.bangsi	Dorsal	0.46	0.46	1287.88
374501	H.l.bangsi	Dorsal	0.56	0.61	1136.36
374501	H.l.bangsi	Ventral	0.50	0.51	1136.36
374501	H.l.bangsi	Dorsal	0.57	0.62	1136.36
374501	H.l.bangsi	Ventral	0.51	0.44	1136.36
387781	H.l.bangsi	Dorsal	0.52	0.60	1666.67
387781	H.l.bangsi	Ventral	0.42	0.44	1666.67
387782	H.l.bangsi	Ventral	0.37	0.54	1363.64
387782	H.l.bangsi	Ventral	0.36	0.68	1363.64
387783	H.l.bangsi	Dorsal	0.54	0.58	1515.15
387783	H.l.bangsi	Dorsal	0.59	0.45	1515.15
387783	H.l.bangsi	Ventral	0.44	0.52	1515.15
387784	H.l.bangsi	Dorsal	0.58	0.58	1515.15
387784	H.l.bangsi	Ventral	0.45	0.48	1515.15
387784	H.l.bangsi	Ventral	0.44	0.48	1515.15
387785	H.l.bangsi	Dorsal	0.62	0.62	1515.15
387785	H.l.bangsi	Ventral	0.35	0.52	1515.15
387785	H.l.bangsi	Dorsal	0.59	0.62	1515.15
387786	H.l.bangsi	Dorsal	0.56	0.49	1515.15
387786	H.l.bangsi	Ventral	0.38	0.57	1515.15
387787	H.l.bangsi	Ventral	0.39	0.58	1590.91
387787	H.l.bangsi	Dorsal	0.52	0.47	1590.91
387788	H.l.bangsi	Dorsal	0.60	0.54	1515.15
387788	H.l.bangsi	Ventral	0.54	0.63	1515.15
387791	H.l.bangsi	Ventral	0.44	0.63	2621.21
387791	H.l.bangsi	Dorsal	0.55	0.75	2621.21
447220	H.l.brunneiceps	Dorsal	0.54	0.65	1981.00
447219	H.l.brunneiceps	Dorsal	0.57	0.47	2530.00
468482	H.l.brunneiceps	Dorsal	0.59	0.54	2500.00
447220	H.l.brunneiceps	Ventral	0.37	0.53	1981.00
447219	H.l.brunneiceps	Ventral	0.41	0.50	2530.00
468482	H.l.brunneiceps	Ventral	0.45	0.71	2500.00
412379	H.l.leucophrys	Dorsal	0.46	0.56	1829.00

447222	H.l.leucophrys	Dorsal	0.46	0.49	1859.00
412381	H.l.leucophrys	Dorsal	0.48	0.54	1829.00
436754	H.l.leucophrys	Dorsal	0.48	0.66	2400.00
412377	H.l.leucophrys	Dorsal	0.48	0.56	1829.00
374497	H.l.leucophrys	Dorsal	0.48	0.50	1676.00
427162	H.l.leucophrys	Dorsal	0.48	0.77	2591.00
447218	H.l.leucophrys	Dorsal	0.49	0.61	2896.00
436753	H.l.leucophrys	Dorsal	0.49	0.71	1768.00
436745	H.l.leucophrys	Dorsal	0.49	0.57	2728.00
447221	H.l.leucophrys	Dorsal	0.50	0.62	1859.00
447223	H.l.leucophrys	Dorsal	0.51	0.67	2134.00
447225	H.l.leucophrys	Dorsal	0.52	0.64	2560.00
436746	H.l.leucophrys	Dorsal	0.53	0.59	2728.00
447226	H.l.leucophrys	Dorsal	0.53	0.59	2560.00
447227	H.l.leucophrys	Dorsal	0.53	0.61	2591.00
427158	H.l.leucophrys	Dorsal	0.53	0.66	1981.00
427154	H.l.leucophrys	Dorsal	0.53	0.56	1981.00
256231	H.l.leucophrys	Dorsal	0.54	0.63	1829.00
427156	H.l.leucophrys	Dorsal	0.54	0.69	1981.00
427159	H.l.leucophrys	Dorsal	0.55	0.65	1981.00
427161	H.l.leucophrys	Dorsal	0.56	0.85	2591.00
447217	H.l.leucophrys	Dorsal	0.57	0.60	2896.00
447228	H.l.leucophrys	Dorsal	0.57	0.61	3200.00
436748	H.l.leucophrys	Dorsal	0.57	0.62	2728.00
374498	H.l.leucophrys	Dorsal	0.58	0.59	1829.00
427155	H.l.leucophrys	Dorsal	0.58	0.60	1981.00
447224	H.l.leucophrys	Dorsal	0.58	0.60	1713.00
374496	H.l.leucophrys	Dorsal	0.58	0.64	1676.00
427157	H.l.leucophrys	Dorsal	0.59	0.51	1981.00
447216	H.l.leucophrys	Dorsal	0.59	0.66	2896.00
256228	H.l.leucophrys	Dorsal	0.59	0.55	2073.00
436751	H.l.leucophrys	Dorsal	0.60	0.63	1768.00
436752	H.l.leucophrys	Dorsal	0.61	0.61	1768.00
256230	H.l.leucophrys	Dorsal	0.61	0.47	2438.00
256227	H.l.leucophrys	Dorsal	0.61	0.62	2743.00
256229	H.l.leucophrys	Dorsal	0.61	0.57	2530.00
436747	H.l.leucophrys	Dorsal	0.61	0.59	2728.00
412380	H.l.leucophrys	Dorsal	0.62	0.60	1829.00
403137	H.l.leucophrys	Dorsal	0.64	0.57	2150.00
427160	H.l.leucophrys	Dorsal	0.65	0.57	1981.00
436749	H.l.leucophrys	Dorsal	0.65	0.76	2728.00
403138	H.l.leucophrys	Dorsal	0.68	0.57	2200.00
256226	H.l.leucophrys	Dorsal	0.71	0.60	2743.00
436754	H.l.leucophrys	Ventral	0.24	0.57	2400.00
447225	H.l.leucophrys	Ventral	0.29	0.52	2560.00

412380	H.l.leucophrys	Ventral	0.33	0.48	1829.00
447224	H.l.leucophrys	Ventral	0.35	0.44	1713.00
427157	H.l.leucophrys	Ventral	0.35	0.37	1981.00
447223	H.l.leucophrys	Ventral	0.36	0.66	2134.00
436753	H.l.leucophrys	Ventral	0.37	0.61	1768.00
412377	H.l.leucophrys	Ventral	0.37	0.59	1829.00
447217	H.l.leucophrys	Ventral	0.38	0.58	2896.00
412381	H.l.leucophrys	Ventral	0.38	0.43	1829.00
436748	H.l.leucophrys	Ventral	0.39	0.50	2728.00
403137	H.l.leucophrys	Ventral	0.39	0.51	2150.00
427161	H.l.leucophrys	Ventral	0.39	0.64	2591.00
447222	H.l.leucophrys	Ventral	0.39	0.51	1859.00
447218	H.l.leucophrys	Ventral	0.40	0.50	2896.00
427156	H.l.leucophrys	Ventral	0.40	0.50	1981.00
447226	H.l.leucophrys	Ventral	0.40	0.49	2560.00
403138	H.l.leucophrys	Ventral	0.41	0.61	2200.00
427160	H.l.leucophrys	Ventral	0.41	0.54	1981.00
447221	H.l.leucophrys	Ventral	0.41	0.58	1859.00
436752	H.l.leucophrys	Ventral	0.42	0.50	1768.00
427158	H.l.leucophrys	Ventral	0.42	0.51	1981.00
374497	H.l.leucophrys	Ventral	0.42	0.51	1676.00
436745	H.l.leucophrys	Ventral	0.42	0.50	2728.00
256229	H.l.leucophrys	Ventral	0.42	0.52	2530.00
427159	H.l.leucophrys	Ventral	0.43	0.63	1981.00
427155	H.l.leucophrys	Ventral	0.43	0.63	1981.00
447228	H.l.leucophrys	Ventral	0.43	0.55	3200.00
447227	H.l.leucophrys	Ventral	0.44	0.59	2591.00
436751	H.l.leucophrys	Ventral	0.44	0.48	1768.00
427162	H.l.leucophrys	Ventral	0.44	0.58	2591.00
436749	H.l.leucophrys	Ventral	0.44	0.72	2728.00
412379	H.l.leucophrys	Ventral	0.44	0.56	1829.00
427154	H.l.leucophrys	Ventral	0.45	0.71	1981.00
374498	H.l.leucophrys	Ventral	0.46	0.43	1829.00
256228	H.l.leucophrys	Ventral	0.47	0.53	2073.00
436746	H.l.leucophrys	Ventral	0.47	0.56	2728.00
256231	H.l.leucophrys	Ventral	0.48	0.63	1829.00
374496	H.l.leucophrys	Ventral	0.49	0.59	1676.00
256227	H.l.leucophrys	Ventral	0.50	0.55	2743.00
256230	H.l.leucophrys	Ventral	0.51	0.53	2438.00
436747	H.l.leucophrys	Ventral	0.52	0.54	2728.00
447216	H.l.leucophrys	Ventral	0.55	0.63	2896.00
256226	H.l.leucophrys	Ventral	0.63	0.63	2743.00
369775	H.l.manastarae	Dorsal	0.50	0.74	1829.00
374487	H.l.manastarae	Dorsal	0.55	0.59	1676.00
374487	H.l.manastarae	Ventral	0.38	0.54	1676.00

369775	H.l.manastarae	Ventral	0.41	0.54	1829.00
